# A Mutation-Related Long Noncoding RNA Signature of Genome Instability Predicts Immune Infiltration and Hepatocellular Carcinoma Prognosis

**DOI:** 10.3389/fgene.2021.779554

**Published:** 2021-11-22

**Authors:** Jianhua Wu, Xueting Ren, Nan Wang, Ruina Zhou, Mengsha Chen, Yifan Cai, Shuai Lin, Hao Zhang, Xin Xie, Chengxue Dang, Shuqun Zhang, Zhangjian Zhou

**Affiliations:** ^1^ Department of Oncology, The Second Affiliated Hospital of Xi’an Jiaotong University, Xi’an, China; ^2^ Department of Anesthesia, The Second Affiliated Hospital of Xi’an Jiaotong University, Xi’an, China; ^3^ Department of Surgical Oncology, The First Affiliated Hospital of Xi’an Jiaotong University, Xi’an, China; ^4^ Department of Nuclear Medicine, The First Affiliated Hospital of Xi’an Jiaotong University, Xi’an, China

**Keywords:** genomic instability, long non-coding RNAs, hepatocellular carcinoma, prognosis, immune infiltration

## Abstract

**Background:** Long noncoding RNAs (lncRNAs) have been discovered to play a regulatory role in genomic instability (GI), which participates in the carcinogenesis of various cancers, including hepatocellular carcinoma (HCC). We endeavored to establish a GI-derived lncRNA signature (GILncSig) as a potential biomarker and explore its impact on immune infiltration and prognostic significance.

**Methods:** Combining expression and somatic mutation profiles from The Cancer Genome Atlas database, we identified GI-related lncRNAs and conducted functional analyses on co-expressed genes. Based on Cox regression analysis, a GILncSig was established in the training cohort (*n* = 187), and an independent testing patient cohort (*n* = 183) was used to validate its predictive ability. Kaplan-Meier method and receiver operating characteristic curves were adopted to evaluate the performance. The correlation between GI and immune infiltration status was investigated based on the CIBERSORT algorithm and single sample gene set enrichment analysis. In addition, a comprehensive nomogram integrating the GILncSig and clinicopathological variables was constructed to efficiently assess HCC patient prognosis in clinical applications.

**Results:** A total of 88 GI-related lncRNAs were screened out and the functional analyses indicated diversified effects on HCC progression. The GILncSig was established using four independent lncRNAs (AC116351.1, ZFPM2-AS1, AC145343.1, and MIR210HG) with significant prognostic value (*p* < 0.05). Following evaluation with the GILncSig, low-risk patients had significantly better clinical outcomes than high-risk patients in the training cohort (*p* < 0.001), which was subsequently validated in the independent testing cohort. High-risk group exhibited more immunocyte infiltration including B cells memory, macrophages M0 and neutrophils and higher expression of HLA gene set and immune checkpoint genes. Compared to existing HCC signatures, the GILncSig showed better prognosis predictive performance [area under the curve (AUC) = 0.709]. Furthermore, an integrated nomogram was constructed and validated to efficiently and reliably evaluate HCC patient prognosis (3-years survival AUC = 0.710 and 5-years survival AUC = 0.707).

**Conclusion:** The GILncSig measuring GI and impacting immune infiltration serves as a potential biomarker and independent predictor of HCC patient prognosis. Our results highlight further investigation of GI and HCC molecular mechanisms.

## Introduction

Hepatocellular carcinoma (HCC), or malignant hepatoma, has become the most common primary liver malignant tumor, accounting for 7% of all cancers globally (Budny et al., 2017). Despite advances in medical, locoregional, and surgical therapies, the clinical prognosis of HCC is not satisfactory, and its mortality rate remains high (Hartke et al., 2017). The key factors contributing to the development of HCC include viral hepatitis B and C, cirrhosis, fatty liver disease, diabetes, alcohol, aflatoxin, and aristolochic acid (Yang et al., 2019). It is widely acknowledged that the pathogenesis of HCC involves genetic and epigenetic changes, but the molecular mechanisms remain unclear (Ogunwobi et al., 2019). Thus far, scholars have focused on prognostic biomarkers and molecular risk models to better predict HCC patient prognosis and elucidate HCC carcinogenesis (Wang et al., 2019a; Zhang et al., 2020). However, there are several limitations to these studies, including small sample size and lack of functional or mechanical analyses. Thus, there is an urgent need to utilize comprehensive methods to identify potential biomarkers and predict HCC prognosis in clinical management.

Genomic instability (GI) has been recognized as a leading factor in carcinogenesis and a hallmark of cancer (Negrini et al., 2010). Accumulation of GI can be lethal to cells and is correlated with poor prognosis (Andor et al., 2017). Although the cellular mechanisms of GI are not fully understood, replication damage and transcriptional regulation have been recognized to play critical roles (Ferguson et al., 2015; Tubbs and Nussenzweig, 2017). Therefore, scholars have utilized relevant molecular signatures to quantify GI in cancers. For example, Vacher et al. studied 103 bladder cancer cases and identified a palindromic non-coding mutation signature of somatic GI (Vacher et al., 2020). A GI-derived three-microRNA (miRNA) signature in breast cancer constructed by Bao et al. was found to be significantly associated with unfavorable prognosis (Bao et al., 2021).

Long noncoding RNAs (lncRNAs) are a group of non-coding RNAs with more than 200 nucleotides that can regulate the products of gene expression in various cell activities and biological processes and are involved in different types of cancers (Paraskevopoulou and Hatzigeorgiou, 2016; Bhan et al., 2017). Increasing evidence has revealed the significant role of lncRNAs in the maintenance of gene stability via multiple pathways (Oliva-Rico and Herrera, 2017; Thapar, 2018). Kristen et al. identified a novel lncRNA MANCR that was functionally associated with genomic stability, the depletion of which led to DNA damage and cell cycle dysregulation (Tracy et al., 2018). Another subsequent study by Mahmoud et al. stressed the contributions of lncRNA NORAD and the NORAD-PUMILIO axis in the genome maintenance of mammalian cells (Elguindy et al., 2019). However, additional GI-related lncRNAs remain unidentified, and their clinical significance as potential biomarkers and treatment targets for HCC patients requires further investigation.

Hence, in this study, we aimed to identify GI-related lncRNAs by combining somatic mutation and expression profiles based on The Cancer Genome Atlas (TCGA) database and develop a GI-derived lncRNA signature (GILncSig) to quantify GI in HCC and help to predict HCC patient prognosis. Besides, we analyzed the immunocytes infiltration, immune-related pathways and expression profiles to explore the association between the GILncSig and immune status. In addition, a comprehensive nomogram was established by integrating clinical variables and the GILncSig to assess clinical outcomes and more efficiently guide patient management.

## Methods

### Data Collection

The expression profile data and clinical information of HCC patients were extracted from TCGA (https://portal.gdc.cancer.gov/). A total of 371 expression cases in 424 files and 377 clinical cases of HCC were obtained, including 50 normal and 374 tumor tissues with mRNA and lncRNA profiles. In addition, 375 somatic mutation data were downloaded from TCGA. A flow chart showed all procedures in this study in Supplementary Figure S1.

### Identification of GI-Related lncRNAs

Based on mutation profiles, the cumulative somatic mutations in HCC samples were first calculated, and the samples were ranked in descending order. The top 25% of mutation numbers were defined as the genomically unstable (GU)-like samples (*n* = 93), and the bottom 25% were defined as genomically stable (GS)-like samples (*n* = 90). The differentially expressed lncRNAs between the GS and GU groups were defined as GI-related lncRNAs.

Hierarchical cluster analyses were conducted for all samples, and we explored the association between mutation conditions and clusters with different gene stabilities. In addition, according to the Pearson correlation coefficients, the top 10 mRNAs that varied with the GI-related lncRNAs were selected, and a co-expression network was constructed. Furthermore, to comprehend their potential functions in GI development, we conducted functional enrichment analyses, including Kyoto Encyclopedia of Genes and Genomes (KEGG) pathways and Gene Ontology (GO) terms.

### Establishment of the GILncSig

First, all HCC cases with expression profiles and clinical information were randomly divided into two equal groups, a training group (*n* = 187) and a testing group (*n* = 183), for the construction and validation of the GILncSig. The chi-square test was used to demonstrate that there were no significant differences between the two groups. In the training group, univariate Cox analysis was performed to explore the GI-related lncRNAs associated with overall survival, and multivariate Cox analysis identified lncRNAs with independent prognostic value. Combining the results of the Cox regression analysis and the expression profiles of GI-related lncRNAs, a GILncSig with linear risk score formula was established, which applies to all HCC samples and can be calculated as follows:

$$\mathrm{GILncSig}=\sum \left(\mathrm{expression}\;\mathrm{of}\;\ln\mathrm{cRN}A_{n}\times \beta_{n}\right)$$

where n represents the number of independent prognostic lncRNAs, and β represents the regression coefficients from the Cox regression analyses, weighing the value of each lncRNA in the formula.

Then, all HCC samples in the training and testing groups were assigned risk scores and classified into high-risk and low-risk groups by the cutoff of the group median risk score. Log-rank tests and the Kaplan-Meier method were then adopted to verify the predictive ability of the GILncSig, and the performance was further evaluated using receiver operating characteristic (ROC) curves.

### Immune Infiltration Analysis

Immunocytes and related pathways were analyzed by Single Sample Gene Set Enrichment Analysis (ssGSEA) with the “GSVA” package and the infiltration levels of all HCC samples were evaluated with the “estimate” package by R software. Furthermore, we speculated the quantity of 22 immunocyte subtypes in each HCC sample by the corresponding expression signatures of the 22 immunocytes with the CIBERSORT algorithm (Newman et al., 2015). To explore the relationship between GI and immune infiltration, the differential immune fractions between high-risk and low-risk groups were compared by the Wilcoxon test and exhibited with the “vioplot” package. In addition, we compared other immunity profiles between the two groups, including the expression of immune checkpoint genes and human leukocyte antigen (HLA) genes.

### Construction and Evaluation of the Nomogram

A comprehensive nomogram for predicting the survival probability of HCC patients was built by integrating the GILncSig and clinicopathologic variables, such as age, grade, and stage. Based on the Cox regression analyses, the nomogram weighing all predictive variables computed the total points of HCC patients, which could predict their 3- and 5-years survival probability. The higher the score, the worse the prognosis. In addition, Harrell’s C-index and 3- and 5-years calibration curves were generated to assess the predictive performance. Furthermore, based on the nomogram scores of all HCC samples, 3- and 5-years ROC curves and survival analyses were performed to evaluate the reliability and feasibility of the nomogram in clinical applications.

### Statistical Analysis

R (v.4.0.2; The R Foundation, Vienna, Austria) and Excel (Microsoft Corporation, Redmond, WA United States) software were used to conduct all statistical analyses with flexible statistical methods. *p* < 0.05 was set as statistically significant in most parts of our study.

## Results

### Identification of GI-Related lncRNAs in HCC

**FIGURE 1:**
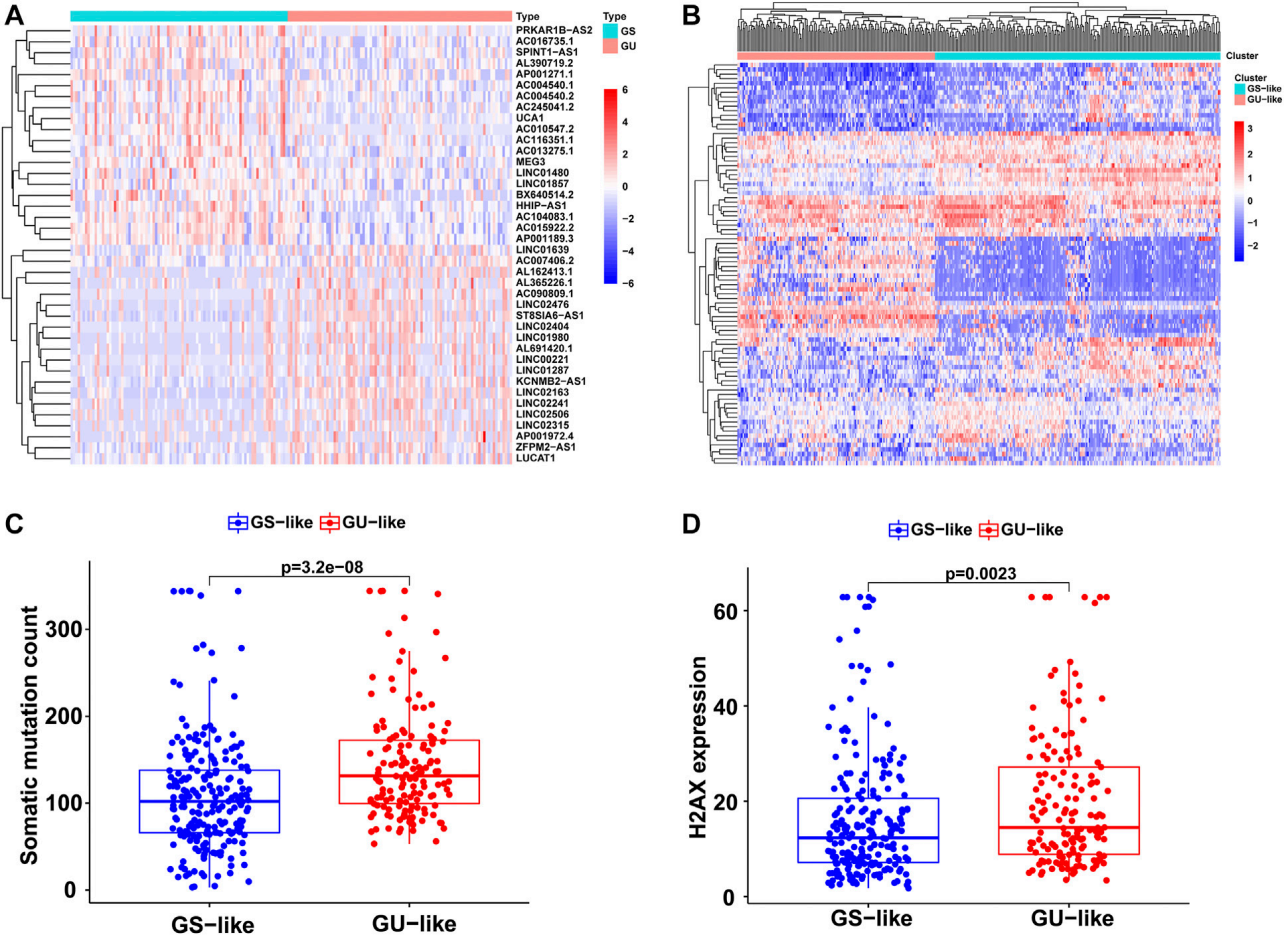
Identification of the GI-related lncRNAs in HCC. **(A)** The top 20 most differentially expressed lncRNAs between the GS and GU group. **(B)** Unsupervised clustering of 374 HCC samples based on the 88 GI-related lncRNAs. The red cluster is the GU-like group and the blue cluster is the GS-like group. **(C)** Boxplots of somatic mutations between the GU-like group and GS-like group. **(D)** Boxplots of H2AX expression level between the GU-like group and GS-like group.

Based on cumulative somatic mutations, all HCC samples from TCGA were classified into the GU-like group of the top 25% of mutation numbers and the GS-like group of the bottom 25% of mutation numbers. Differential expression analysis showed that a total of 88 lncRNAs were significantly differentially expressed between the two groups with a false discovery rate-adjusted *p*-value < 0.05. Compared to the GS-like group, 56 lncRNAs were upregulated and 32 lncRNAs were downregulated in the GU-like group. The top 20 most differentially expressed lncRNAs were selected using fold change and are shown in a heatmap (Figure 1A).

Based on the 88 GI-related lncRNAs, unsupervised hierarchical clustering classified all 374 HCC samples into two groups: GS-like (*n* = 221) and GU-like (*n* = 153; Figure 1B). There were significantly higher somatic mutation counts in the GU-like group than in the GS-like group (median value: 131.5 *vs*. 102; *p* < 0.001; Figure 1C). Meanwhile, the expression of H2AX, an identified driver gene associated with gene instability and cancer onset, was compared between the two groups. The expression of H2AX was significantly higher in the GU-like group than in the GS-like group (*p* < 0.01; Figure 1D).

**FIGURE 2:**
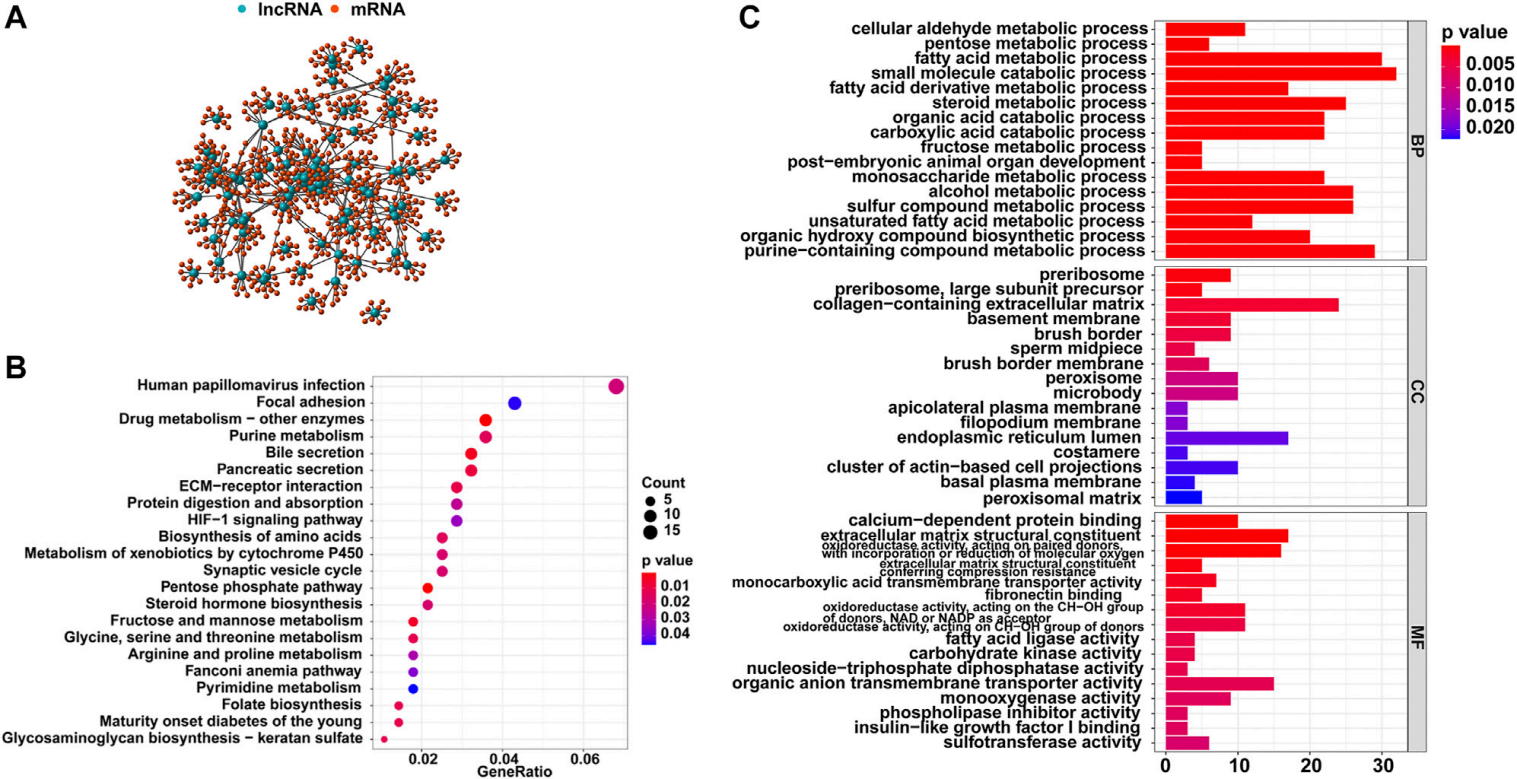
Functional analysis of the GI-related lncRNAs in HCC. **(A)** Co-expression network of GI-related lncRNAs and top 10 relevant mRNAs. **(B)** KEGG enrichment analysis for the co-expressed genes. **(C)** GO functional analysis for the co-expressed genes.

Then, the top 10 mRNAs relevant to each GI-related lncRNA were selected, and an mRNA-lncRNA co-expression network was established (Figure 2A). Furthermore, functional analyses were performed on these mRNAs to explore the potential functions of the 88 lncRNAs in GI occurrence. KEGG analysis revealed that most genes relevant to the lncRNAs were significantly enriched in 22 pathways, including pyrimidine metabolism, purine metabolism, and folate biosynthesis, which participate in the synthesis of nucleotides and may affect genomic stability (Figure 2B). As for the GO analysis shown in Figure 2C, the GI-related mRNAs were significantly linked to biological processes involved in the metabolism of genetic material, including purine-containing compound metabolic processes and small molecule catabolic processes. The other significant enrichment terms revealed in the cellular component and molecular function analyses indicated the probable mechanisms in the formation and development of GI.

### Establishment of the GILncSig for Prognosis Prediction

**FIGURE 3:**
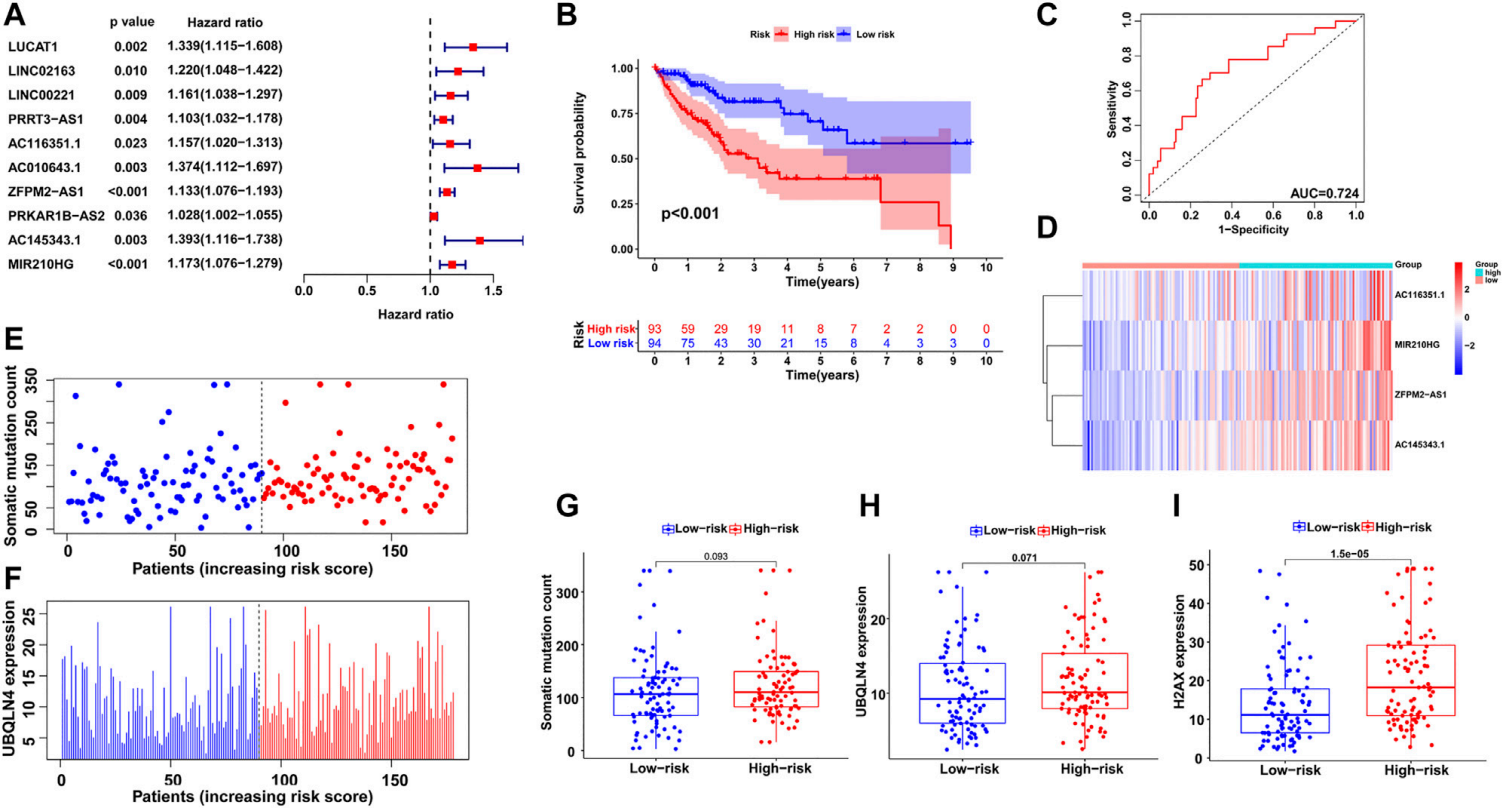
Establishment of GILncSig for prognosis prediction in the training group. **(A)** Univariate Cox regression analysis of the GI-related lncRNAs associated with overall survival in the training group. **(B)** Kaplan–Meier survival analysis of high-risk and low-risk groups predicted by the GILncSig. **(C)** ROC curve to evaluate the performance of the GILncSig. **(D)** The expression pattern of the GILncSig in the training group. **(E)** Distribution of somatic mutations with increasing risk score. **(F)** UBQLN4 expression with increasing risk score. The boxplots of the distribution of somatic mutations, **(G)** UBQLN4 expression **(H)** and H2AX expression **(I)** between the high-risk and low-risk groups in the training group.

All HCC cases were randomly and equally divided into a training group (*n* = 187) and a testing group (*n* = 183), and a chi-square test showed that there were no significant differences in clinicopathological features between the two groups (Table 1). To determine the prognostic value of GI-related lncRNAs, univariate Cox regression analysis was conducted among the training group, and 10 lncRNAs were found to be significantly associated with overall survival (*p* < 0.05; Figure 3A). Multivariate Cox analysis identified four lncRNAs with independent values: AC116351.1, ZFPM2-AS1, AC145343.1, and MIR210HG. Based on the Cox analysis and expression profiles of HCC patients, a GILncSig was established with a linear risk score formula combining the four independent GI-related lncRNAs weighted by coefficients from the multivariate analysis. Hence, the risk scores of all HCC patients can be calculated as follows: GILncSig score = (0.1594 × expression level of AC116351.1) + (0.1189 × expression level of ZFPM2-AS1) + (0.2247 × expression level of AC145343.1) + (0.1092 × expression level of MIR210HG). All coefficients of the four lncRNAs were positive, implying that they were risk factors for HCC prognosis.

**TABLE 1:** Clinical information of three HCC patients sets in this study.

Covariates		Training set (*n* = 187)	Testing set (*n* = 183)	TCGA set (*n* = 370)	*p* Value
Age(%)	≤65	115(61.5)	117(63.93)	232(62.7)	0.706
	>65	72(38.5)	66(36.07)	138(37.3)	
Gender(%)	FEMALE	62(33.16)	59(32.24)	121(32.7)	0.939
	MALE	125(66.84)	124(67.76)	249(67.3)	
Grade(%)	G1-2	117(62.57)	115(62.84)	232(62.7)	0.985
	G3-4	68(36.36)	65(35.52)	133(35.95)	
	unknow	2(1.07)	3(1.64)	5(1.35)	
Stage(%)	I-II	130(69.52)	126(68.85)	256(69.19)	0.999
	III-IV	46(24.6)	44(24.04)	90(24.32)	
	unknow	11(5.88)	13(7.1)	24(6.49)	

Then, using the GILncSig, all HCC patients were assigned risk scores in the training set and were subsequently divided into two groups based on the median risk score: a high-risk group with higher scores and a low-risk group with lower scores. Log-rank tests and Kaplan–Meier curves showed that the high-risk group had significantly poorer prognosis than the low-risk group (*p* < 0.001; Figure 3B). An ROC curve was then generated to assess the reliability of the GILncSig, and the area under the curve (AUC) was 0.724 (Figure 3C), indicating good predictive ability. Furthermore, we explored the changes in GILncSig expression, somatic mutation count, and UBQLN4 expression in all HCC patients along with their increasing risk scores in the training group (Figures 3D–F). In the high-risk group, the expression levels of the four lncRNAs were all increased, somatic mutations became more frequent, and the expression level of UBQLN4, a GI-driver gene, was also upregulated compared to the low-risk group. Additionally, two boxplots were drawn to demonstrate the UBQLN4 expression and somatic mutation number trends in patients along with their increasing risk scores (Figures 3G,H). There was a visible increase in the two plots, but it was not statistically significant, which was further verified in the following section. The expression of H2AX was significantly higher in the high-risk group than in the low-risk group, which was consistent with the previous result (*p* < 0.05; Figure 3I).

### Independent Validation of the GILncSig in HCC Datasets

**FIGURE 4:**
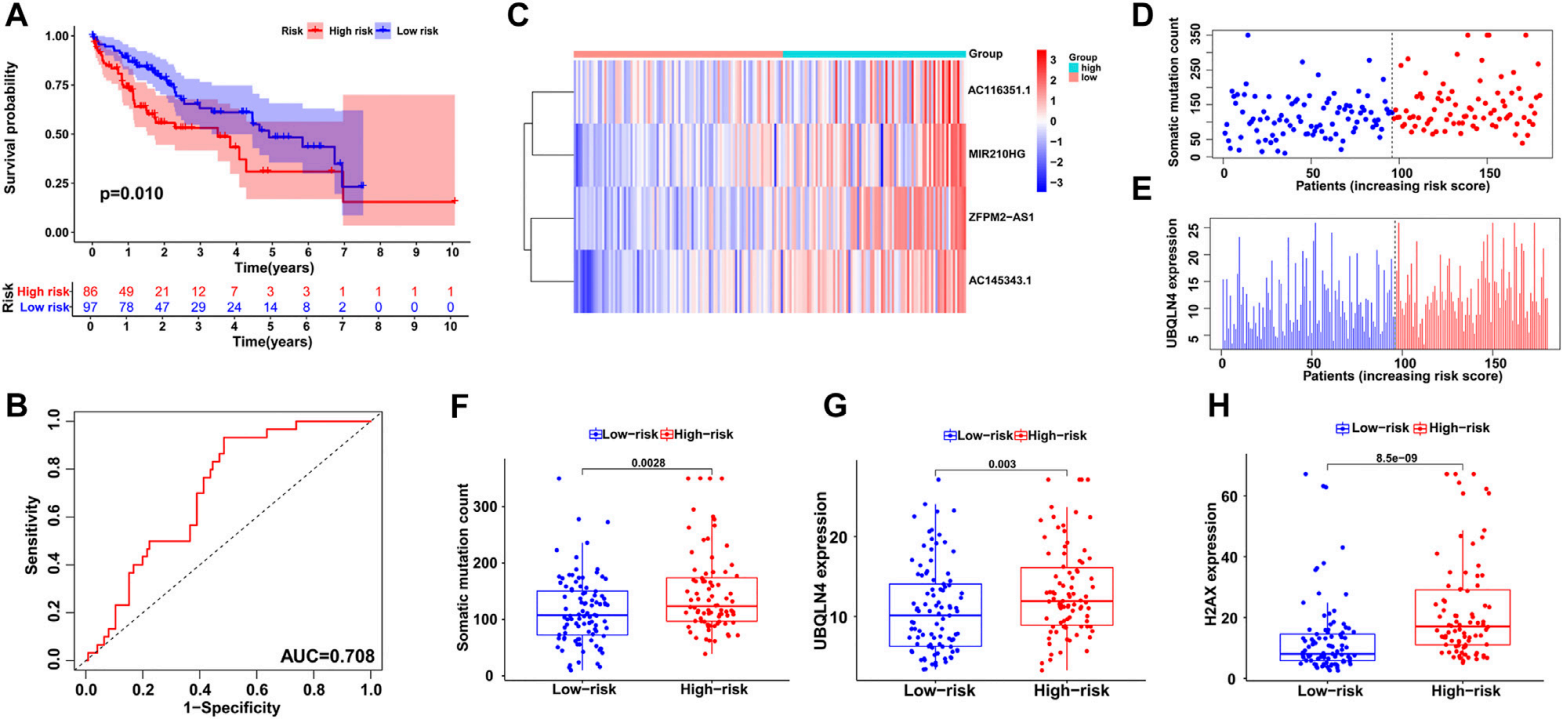
Independent validation of the GILncSig in the testing group. **(A)** Kaplan–Meier survival analysis of high-risk and low-risk groups. **(B)** ROC curve to evaluate the performance of the GILncSig. **(C)** The expression pattern of the GILncSig in the testing group. **(D)** Distribution of somatic mutations with increasing risk score. **(E)** UBQLN4 expression with increasing risk score. The boxplots of the distribution of somatic mutations **(F)**, UBQLN4 expression **(G)** and H2AX expression **(H)** between the high-risk and low-risk groups in the testing group.

To validate the feasibility and reliability of the GILncSig established in the training set, we applied the signature to all HCC patients in the testing set (*n* = 183) and obtained their risk scores for prognosis. The patients were then classified into two groups with different prognosis risks according to the median score, and Kaplan-Meier analysis showed that the high-risk group had significantly poorer clinical outcomes than the low-risk group (*p* < 0.05; Figure 4A). An ROC curve of the GILncSig with an AUC of 0.708 was produced, representing good sensitivity and specificity (Figure 4B). Furthermore, we displayed the patients with risk scores in increasing order and analyzed the tendencies of GILncSig expression, somatic mutation count, and UBQLN4 expression in all HCC patients (Figures 4C–E). Similar to the training group, they were all positively associated with risk score, which is further illustrated in Figure 4F and Figure 4G. Somatic mutation count and UBQLN4 expression were both significantly increased in the high-risk group (*p* < 0.01). In addition, H2AX expression was significantly upregulated (*p* < 0.001; Figure 4H).

**FIGURE 5:**
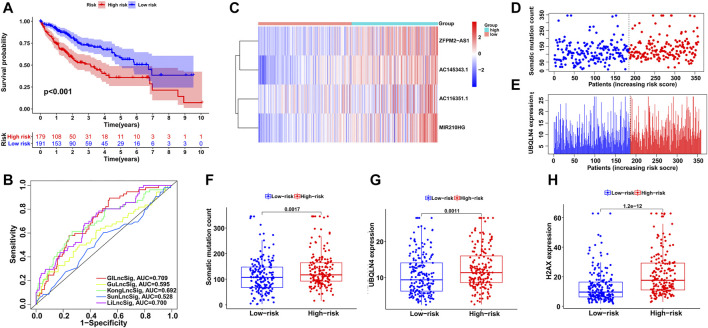
Evaluation of the GILncSig in the TCGA set. **(A)** Kaplan–Meier survival analysis of high-risk and low-risk groups. **(B)** ROC analysis to evaluate the performance of the GILncSig, GuLncSig, KongLncSig, SunLncSig and LiLncSig. **(C)** The expression pattern of the GILncSig in the TCGA set. **(D)** Distribution of somatic mutations with increasing risk score. **(E)** UBQLN4 expression with increasing risk score. The boxplots of the distribution of somatic mutations **(F)**, UBQLN4 expression **(G)** and H2AX expression **(H)** between the high-risk and low-risk groups in the testing group.

Next, we utilized all HCC cases from TCGA to examine the performance of the GILncSig and obtained similar but more significant results. After all patients were assigned risk scores and divided into high- and low-risk groups, log-rank tests and Kaplan–Meier curves showed significant survival differences between the two groups (*p* < 0.001; Figure 5A). ROC curve analysis showed the reliability of the signature with an AUC of 0.709 (Figure 5B). As shown in Figures 5C–E, the distributions of GILncSig expression, somatic mutation count, and UBQLN4 expression in all HCC patients along with their increasing scores became more evident. The three boxplots verified the trends of increasing mutation counts and expression levels of UBQLN4 and H2AX with statistical significance (*p* < 0.001, Figure 5F; *p* < 0.001, Figure 5G; and *p* < 0.001, Figure 5H, respectively).

### Comparison of Predictive Ability of lncRNA Signatures

Subsequently, we compared the GILncSig in this study and 4 other lncRNA signatures of HCC prognosis from previous studies: the 6-lncRNA signature from Gu’s study (mentioned as GuLncSig) (Gu et al., 2019), the 2-lncRNA signature from Kong’s study (mentioned as KongLncSig) (Kong et al., 2020), the 5-lncRNA signature from Sun’s study (mentioned as SunLncSig) (Sun et al., 2019) and the 11-lncRNA signature from Li’s study (mentioned as LiLncSig) (Li et al., 2020). Based on the same cohort from TCGA, we applied these lncRNA signatures to all HCC patients to evaluate their prognosis, and the performances of the signatures were compared by ROC curve analysis. As shown in Figure 5B, the AUC of GILncSig was 0.709, which was higher than that of all four other signatures: GuLncSig (AUC = 0.595), KongLncSig (AUC = 0.692), SunLncSig (AUC = 0.528), and LiLncSig (AUC = 0.700). In addition, the GILncSig consists of 4 fewer lncRNAs than the GuLncSig (6 lncRNAs), SunLncSig (5 lncRNAs), and LiLncSig (11 lncRNAs). These comparisons provided evidence of better performance of the GILncSig in predicting HCC patient prognosis.

### Independent GILncSig Prediction From Other Clinical Factors

**FIGURE 6:**
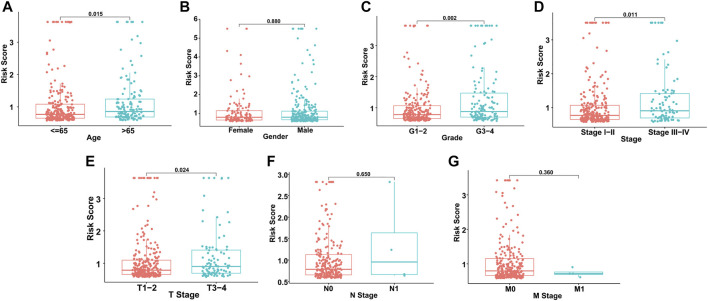
Risk score distribution in different clinical subgroups. **(A)** Age. **(B)** Gender. **(C)** Grade. **(D)** Stage. **(E)** T stage. **(F)** N stage. **(G)** M stage.

**FIGURE 7:**
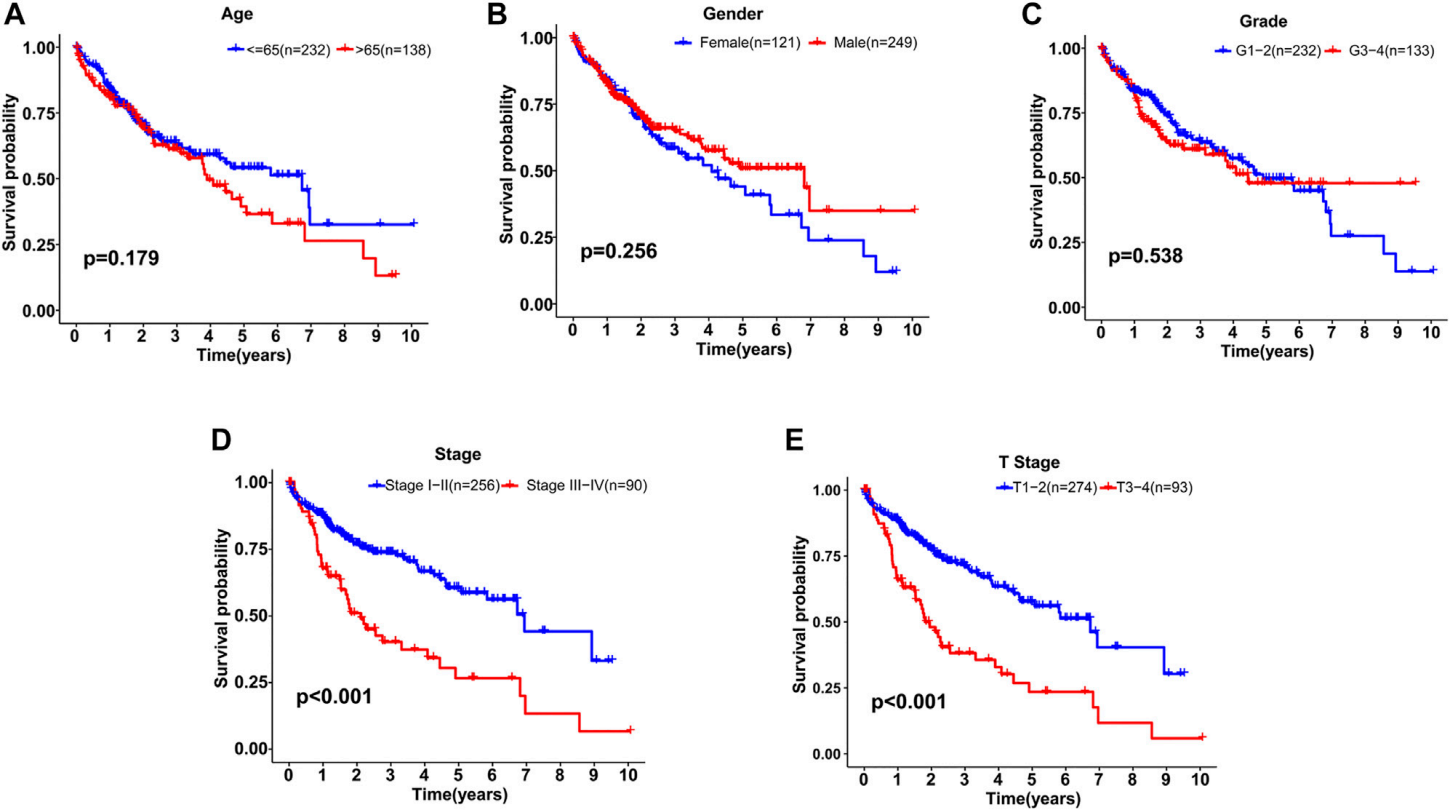
Kaplan–Meier survival analyses of patients with different clinical characteristics. **(A)** Age. **(B)** Gender. **(C)** Grade. **(D)** Stage. **(E)** T stage.

**FIGURE 8:**
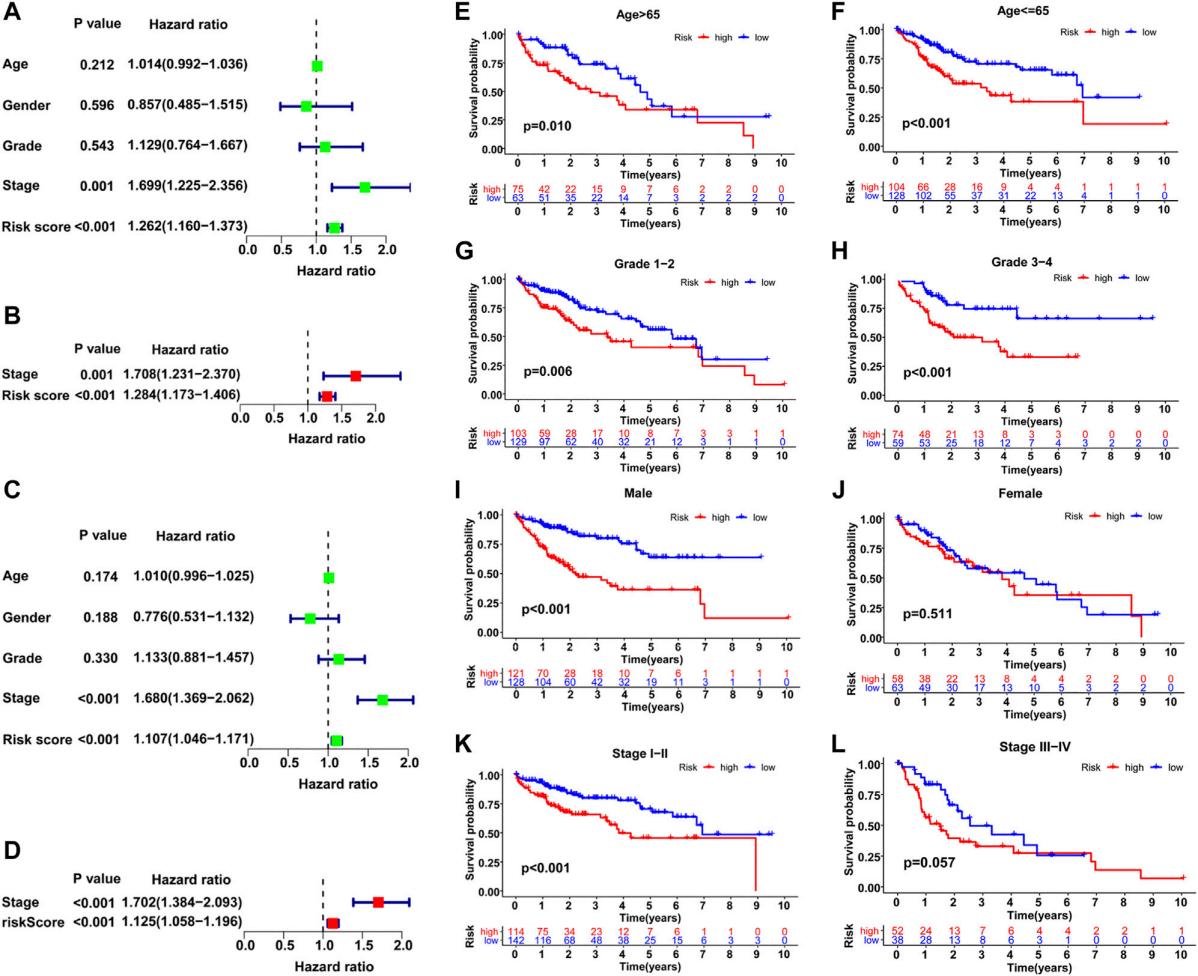
Comparison of the prognostic value of the GILncSig and other clinical variables. Training set **(A, B)**, TCGA set **(C–L)**. **(A–C)** Univariate Cox regression analyses of the OS-related parameters. **(B–D)** Multivariate Cox regression analysis of the OS-related parameters. Stratification analysis and Kaplan–Meier survival analysis in high-risk and low-risk groups for old patients **(E)**, young patients **(F)**, early-grade patients **(G)**, late-grade patients **(H)**, male **(I)**, female **(J)**, early-stage patients **(K)** and late-stage patients **(L)**.

Based on the distribution of risk scores, HCC patients had significantly higher risks in clinical subgroups of >65 (*p* = 0.015), grade 3–grade 4 (*p* = 0.0017), stage III−IV (*p* = 0.011) and T3-4 stage (*p* = 0.024, Figure 6). As clinical characteristics are commonly used in clinical prognosis evaluation, it is necessary to explore the independency and compare the prediction efficiency among the GILncSig risk score and clinical factors. Firstly Kaplan–Meier survival analyses examined the prognostic ability of traditional clinical variables and patients in higher stage or higher T stage showed worse clinical outcome (Figure 7). Within all HCC samples in the training set, we first performed univariate Cox regression analysis to select potential predictors related to overall survival (Figure 8A). Next, using multivariate Cox regression analysis, stage [hazard ratio (HR) = 1.708, 95% confidence interval (CI): 1.231–2.370; *p* < 0.01] and risk score (HR = 1.284, 95% CI: 1.173–1.406; *p* < 0.001) were identified to be independent factors (Figure 8B). In the testing set, the two factors presented similarly good prediction abilities, but only stage showed significant meaning. Furthermore, the Cox analyses among all HCC cases in the database validated the results, as stage (HR = 1.702, 95% CI: 1.384–2.093; *p* < 0.001) and risk score (HR = 1.125, 95% CI: 1.058–1.196; *p* < 0.001) showed significantly independent prognostic values for HCC prognosis (Figures 8C,D). The results of all Cox analyses are shown in Table 2.

**TABLE 2:** Cox regression analyses of clinical variables and GILncSig risk score associated with overall survival in HCC.

	Cox analysis	HR	95% CI	*p* Value
TCGA set	Univariate			
(*n* = 370)	Age	1.010	0.996–1.025	0.174
	Gender	0.776	0.531–1.132	0.188
	Grade	1.133	0.881–1.457	0.330
	Stage	1.680	1.369–2.062	<0.001
	Risk score	1.107	1.046–1.171	<0.001
	multivariate			
	Stage	1.702	1.384–2.093	<0.001
	Risk score	1.125	1.058–1.196	<0.001
Training set	univariate			
(*n* = 187)	Age	1.014	0.992–1.036	0.212
	Gender	0.857	0.485–1.515	0.596
	Grade	1.129	0.764–1.667	0.543
	Stage	1.699	1.225–2.356	0.001
	Risk score	1.262	1.160–1.373	<0.001
	multivariate			
	Stage	1.708	1.231–2.370	0.001
	Risk score	1.284	1.173–1.406	<0.001
Testing set	univariate			
(*n* = 183)	Age	1.009	0.989–1.030	0.363
	Gender	0.715	0.430–1.190	0.197
	Grade	1.132	0.814–1.573	0.461
	Stage	1.679	1.295–2.176	<0.001
	Risk score	1.040	0.943–1.147	0.435
	multivariate			
	Stage	1.679	1.295–2.176	<0.001

In addition, a stratified analysis was conducted to examine the GILncSig among subgroups with different clinical characteristics. Patients were first divided into two or three groups based on different clinical terms and then classified into high- and low-risk teams within these initial groupings for further survival analysis. As shown in Figures 8E–L, patients with lower risks survived significantly longer than those with higher risks in most clinical subgroups, including >65 (*p* = 0.01), ≤65 (*p* < 0.001), male (*p* < 0.001), grade 1–grade 2 (*p* = 0.006), grade 3–grade 4 (*p* < 0.001), and stage I–II (*p* < 0.001). However, the results in the female and stage III−IV subgroups were not satisfactory, which may be due to the small sample size; the *p* value was only marginally significant in stage III−IV (*p* = 0.057). Overall, the GILncSig was an independent predictor and performed well in the classification of patient prognosis within different clinical subgroups.

### Immune Infiltration Analysis of HCC Samples

**FIGURE 9:**
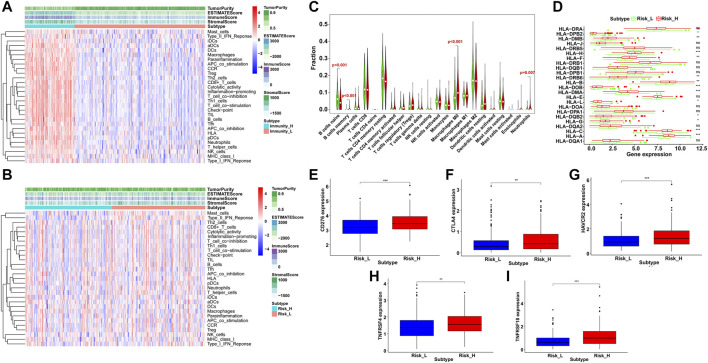
Immune infiltration and profile analysis in HCC. By ssGSEA, immunocyte subtypes and pathways enrichment **(A)** in high- and low-immunity clusters and **(B)** in high- and low-risk groups. The immune score, stromal score, ESTIMATE score and tumor purity were shown in the heatmaps. **(C)** Violin plots of infiltrated immunocyte subtypes between high-risk (red) and low-risk (green) groups. **(D)** Differential expression level of HLA related genes between high-risk (red) and low-risk (green) groups (ns = not significant, *p < 0.05, **p < 0.01, ***p < 0.001). The expression level of immune checkpoint genes between high-risk (red) and low-risk (blue) groups, including CD276 (E), CTLA4 (F), HAVCR2 (G), TNFRSF4 **(H)** and TNFRSF18. (**I**) **p < 0.01, ***p < 0.001).

Based on the result of ssGSEA, all HCC samples were classified into high- and low-immunity clusters and the distribution of 29 immunocyte subtypes and immune related pathways of each HCC sample was shown in a heatmap (Figure 9A). Besides, the immune infiltration level was estimated by immune score, stromal score, ESTIMATE score and tumor purity. The ESTIMATE score was the sum of the former two scores and represented the immune status of the microenvironment. Similarly, the immune fraction distribution and immunity scores of each sample in high- and low-risk groups was shown in Figure 9B. Classified by immunity cluster or scores, HCC patients with higher risks possessed lower survival rate than those with lower risks (Supplementary Figure S2). Then we speculated 22 immunocytes percentage in HCC samples between the two risk groups (Figure 9C). The proportion of B cells memory, macrophages M0 and neutrophils were significantly higher in the high-risk group while B cells naïve were significantly lower (*p* < 0.01), which indicated a higher immune infiltration in the high-risk group.

Furthermore, the immune related expression profiles were compared between the two risk groups. The high-risk group showed a higher expression of HLA gene set than the low-risk group (Figure 9D). In addition, we examined 16 immune checkpoint genes and the expression of five genes were significantly upregulated in the high-risk group, including CD276, CTLA4, HAVCR2, TNFRSF4 and TNFRSF18 (Figures 9E–I), which provided potential immunotherapy targets and indicated a better response to the immune inhibiting reagents in the high-risk group.

### Comprehensive Nomogram to Predict HCC Patient Prognosis

**FIGURE 10:**
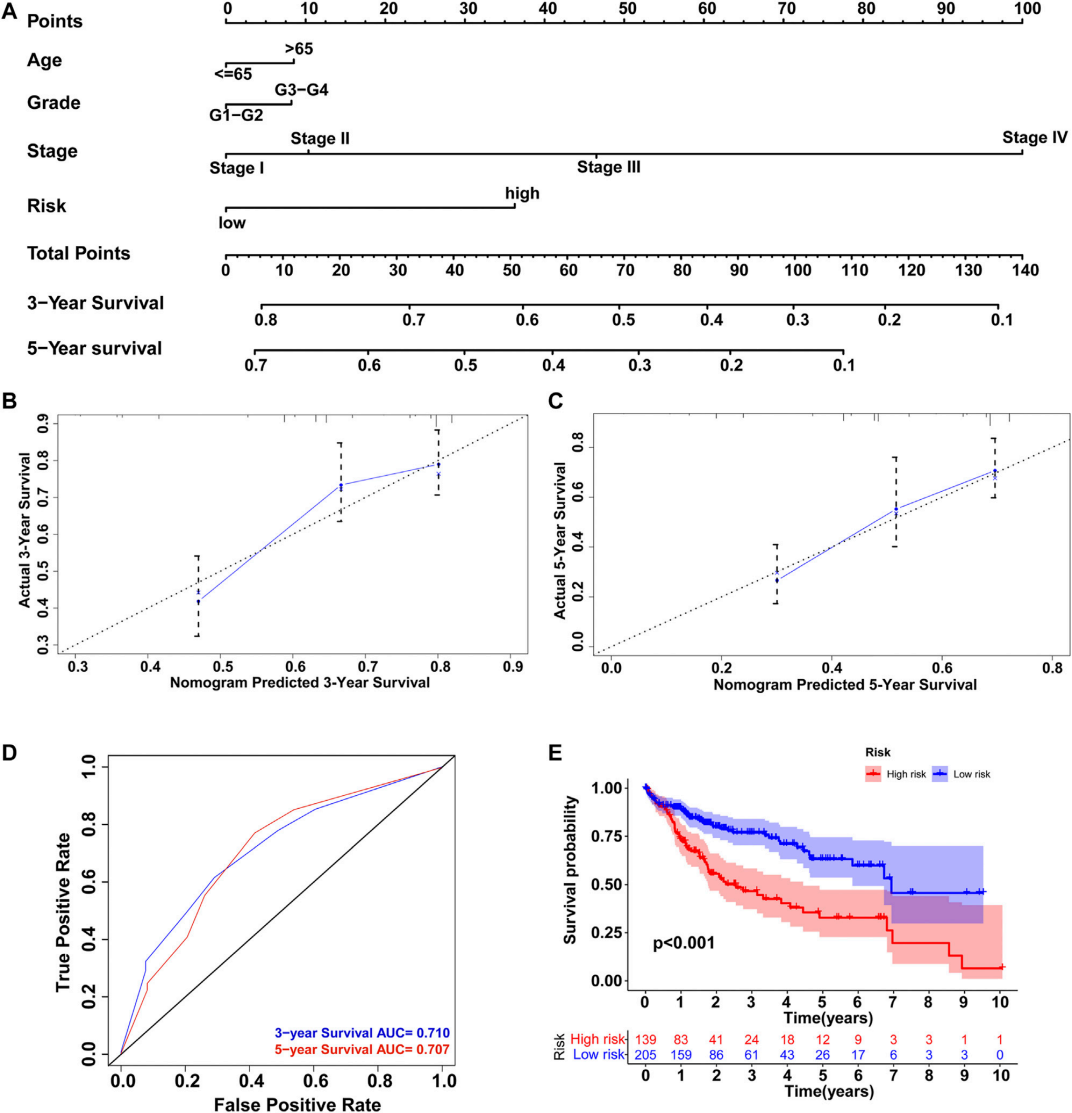
Construction of nomogram to predict the prognosis of HCC patients. **(A)** Comprehensive nomogram integrating the GILncSig and clinicopathological features. Calibration plot of the nomogram model to predict **(B)** 3-years survival and **(C)** 5-years survival. **(D)** ROC curves of the model prediction of 3 and 5-years survival. **(E)** Kaplan–Meier survival analysis of the high-risk and low-risk groups classified by the nomogram.

To develop an efficient prediction tool in clinical practice, a comprehensive nomogram was constructed by integrating the GILncSig and clinicopathological features, including age, grade, and stage (Figure 10A). The nomogram could then assess the 3- and 5-years survival rates of HCC patients based on the total points of the prognostic factors weighed by coefficients. The higher the points, the worse the prognosis. To evaluate the nomogram performance, calibration plots in which the nomogram-predicted survival rate was close to the actual survival of both 3- and 5-years conditions were drawn (Figures 10B,C). Harrell’s concordance index for survival prediction was 0.673 (95% CI: 0.622–0.724). In addition, time-dependent ROC curve analysis of the 3-years (AUC = 0.710) and 5-years (AUC = 0.707) survival predictions was conducted, and the results showed good reliability of the nomogram (Figure 10D). Furthermore, we classified all HCC patients into high-risk and low-risk groups based on the median points produced by the nomogram and performed Kaplan–Meier survival analysis. As shown in Figure 10E, patients with lower risk levels had significantly better clinical outcomes (*p* < 0.001). Therefore, the integrated nomogram was validated as an efficient and reliable tool for evaluating HCC patient prognosis.

## Discussion

In recent decades, considerable efforts have been made to explore the initiation mechanisms and potential treatment methods of HCC (Dimri and Satyanarayana, 2020). Traditional clinicopathological features are still used as predictive tools for HCC prognosis in clinical practice, but molecular risk factors may offer more precise predictions, which could facilitate individualized treatment of HCC patients and help with the allocation of medical resources (Fujiwara et al., 2018; Yin et al., 2019).

Recent studies have discovered that GI plays a vital role in cancer evolution and is related to poor prognosis (Andor et al., 2017; Tubbs and Nussenzweig, 2017). Investigation of colorectal cancer has revealed the contribution of GI in carcinogenesis as early as the premalignant phase through complex mechanisms, including DNA damage and transcriptional mistakes (Grady and Carethers, 2008). Therefore, detectable GI-related molecules have been utilized for the quantification of GI and for further prediction of cancer patient prognosis. Emerging studies have focused on the prognostic value and potential mechanisms of GI-related miRNAs, genes, and relevant signatures in multiple cancers (Vincent et al., 2014; Zhang et al., 2019a), but the role of lncRNAs has been largely neglected thus far. Some recent findings revealed the functions of GI-related lncRNAs, including participation in the DNA damage response, DNA replication, and mitotic and mitochondrial genome maintenance (Lee et al., 2016; Du Mee et al., 2018; Burger et al., 2019). However, the identification and application of GI-related lncRNAs as a means of measuring GI in cancers are new, and the construction of an lncRNA signature to predict HCC patient prognosis requires further exploration.

In this study, we combined somatic mutations with expression profiles and screened 88 GI-related lncRNAs in the TCGA database of HCC patients. KEGG analysis showed that the genes co-expressed with GI-related lncRNAs were enriched in 22 pathways, including pyrimidine metabolism, purine metabolism, Fanconi anemia (FA), and folate biosynthesis, all of which may affect genomic stability. Excessive pyrimidine synthesis over purine results in DNA transversion mutations and genomic signatures (Lee et al., 2018). In addition, studies have shown that the FA pathway guards genomic stability *via* the signaling network of DNA damage repair, and the knockdown of FA genes could impair break end resection and homologous recombination repair (Palovcak et al., 2017; Cai et al., 2020). In addition, GO analysis suggested that the GI-related genes were enriched in various terms, including purine-containing compound metabolic processes and small molecule catabolic processes related to genomic stability.

Furthermore, we selected GI-related lncRNAs with independent prognostic values and established an lncRNA signature (GILncSig) using AC116351.1, ZFPM2-AS1, AC145343.1, and MIR210HG. In a recent study, AC116351.1 showed significant associations with DNA repair and prognostic value in HCC (Zeng et al., 2021). Overexpression of lncRNA ZFPM2-AS1 in HCC tissue was correlated with poorer overall survival, and through *in vitro* functional analysis, ZFPM2-AS1 was found to act as miRNA sponge for promoting HCC cell proliferation, apoptosis, migration, and invasion *via* multiple axes (He et al., 2020; Liu et al., 2020; Zhang et al., 2021). Similarly, MIR210HG is an oncogenic lncRNA that is upregulated in HCC, and its silencing suppresses proliferation, migration, and invasion (Wang et al., 2019b). Little is known about AC145343.1, but the replaced version AC145343.2 was identified as a prognostic factor for glioma mesenchymal transition (Liang et al., 2020). Collectively, these four lncRNAs play vital functions in cancer onset and have shown prognostic value; however, their roles in GI and the combined predictive ability of the established GILncSig remain unknown in previous studies.

Following evaluation with the GILncSig, patients with lower predicted risk levels survived longer than those with higher risk levels in the training set, and the independent internal testing set further validated this result. In contrast to traditional clinical factors, the GILncSig showed comparable or better predictive performance and presented good classification ability within clinical subgroups. In addition, GILncSig expression was significantly associated with somatic mutation counts and expression levels of UBQLN4 and H2AX in all HCC cohorts. UBQLN4, an identified GI driver in multiple cancers, has been found to be overexpressed in aggressive tumors and related to poor outcomes (Jachimowicz et al., 2019; Yu et al., 2020). Similarly, H2AX is involved in GI through DNA damage repair, and its phosphorylated form marks the double-strand break (Seo et al., 2012; Subbiahanadar Chelladurai et al., 2020). Overall, the GILncSig appears to act as a good indicator of both overall survival and GI characteristics of HCC patients. From a therapeutic perspective, the GILncSig provides potential targets for individualized treatment of HCC and further informs medicine resource management concerning personal predicted prognosis.

Besides, the GILncSig associated with somatic mutation would likely to cause a more active immune reaction. We explored the immunocytes and immune related pathways by ssGSEA and estimate the immune microenvironment between the high- and low-risk groups scored by GILncSig. The infiltration of 22 immunocyte subtypes was investigated with the CIBERSORT algorithm and B cells memory, macrophages M0 and neutrophils were more enriched in the high-risk group, consistent with the result of a recent study on immune-related prognostic index in HCC (Hu et al., 2020). High density of IgM+ and CD27− isotype-switched memory B cells was correlated with better survival, which may offer novel therapeutic targets (Zhang et al., 2019b). Macrophages M0 could aggravate HCC development stimulated by the CCAT1/let-7b/HMGA2 pathway (Deng et al., 2020). Similarly, tumor-associated neutrophils promoted HCC progression and could be identified as potential targets for HCC treatment (Zhou et al., 2016; Peng et al., 2020). Furthermore, we compared the expression profiles of immune related genes between the two risk groups and notably, five immune checkpoint genes were upregulated in the high-risk group, including CTLA4, indicating the potential application of the inhibiting agents.

Although this study elucidates the molecular mechanisms of GI in HCC and provides potential biomarkers and efficient evaluation tools for GI and patient prognosis, there are some limitations that require further investigation. Despite the internal validation in the TCGA database, additional large, independent, and complete data sources are needed to verify our findings. We explored the Gene Expression Omnibus database, but the expression and clinical data were inadequate for a complete validation process. In addition, as the identification and function of the GILncSig were analyzed based on bioinformatics methods, experiments involving laboratory measurements and animal models are required in future demonstrations of the GILncSig regulatory mechanisms in HCC development.

In conclusion, we identified a GI-derived lncRNA signature of HCC that may serve as a potential biomarker and independent predictor of HCC prognosis. Furthermore, the comprehensive nomogram integrating the GILncSig and clinical characteristics appeared to efficiently evaluate the overall survival of HCC patients in clinical practice. Our results will likely help to guide further investigations of GI and the molecular mechanisms of HCC.

## Data Availability

The original contributions presented in the study are included in the article/Supplementary Material, further inquiries can be directed to the corresponding authors.

## Supplementary Material

Supplementary Figure S1 A flow chart of the analysis procedures in this study.

Supplementary Figure S2 Kaplan–Meier survival analysis in high-risk and low-risk groups stratified by different immune status. **(A)** High immune cluster. **(B)** Low immune cluster. **(C)** High tumor purity. **(D)** Low tumor purity. **(E)** High ESTIMATE score. **(F)** Low ESTIMATE score. **(G)** High immune score. **(H)** Low immune score. **(I)** High stromal score. **(J)** Low stromal score.
