# Supplementary figures and images for: A Mutation-Related Long Noncoding RNA Signature of Genome Instability Predicts Immune Infiltration and Hepatocellular Carcinoma Prognosis
Source: Front Genet. 2021 Nov 22;12:779554. doi: 10.3389/fgene.2021.779554 (PMC8645863; doi:10.3389/fgene.2021.779554)

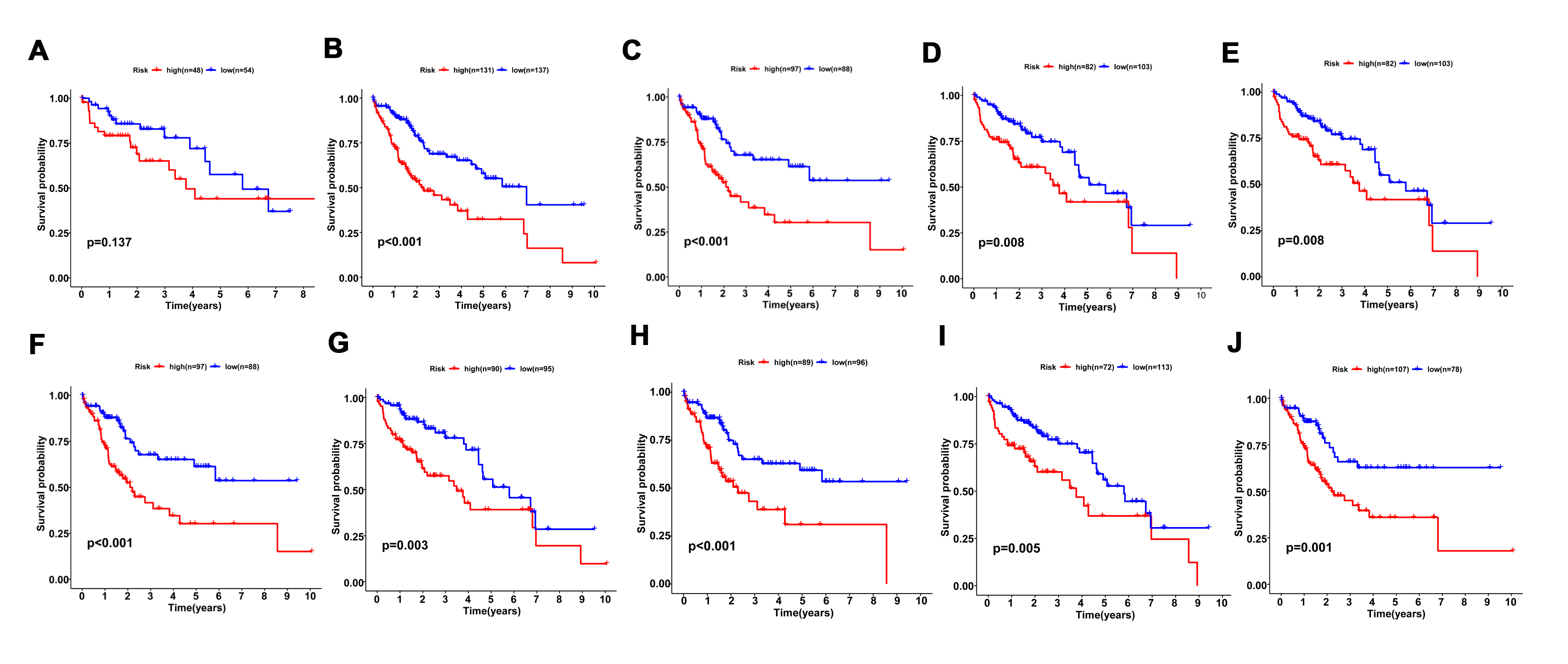

Supplement: Supplementary file 1 [file Image2.TIF]

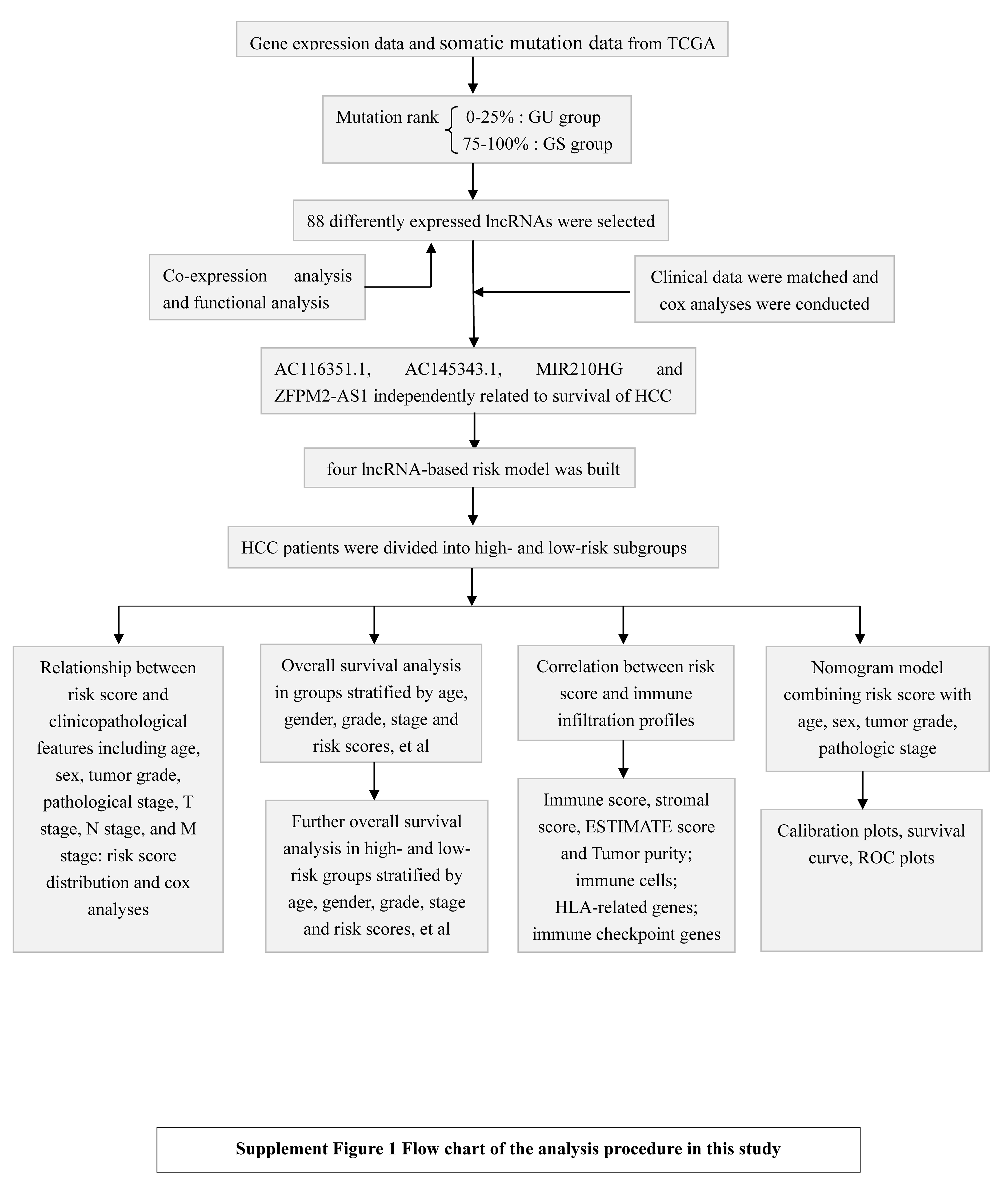

Supplement: Supplementary file 2 [file Image1.TIF]
